# Some Human Anti-Glycan Antibodies Lack the Ability to Activate the Complement System

**DOI:** 10.3390/antib13040105

**Published:** 2024-12-23

**Authors:** Nadezhda Shilova, Alexey Nokel, Alexander Lipatnikov, Nailya Khasbiullina, Yuri Knirel, Ludmila Baidakova, Alexander Tuzikov, Sergei Khaidukov, Polina Obukhova, Stephen Henry, Batozhab Shoibonov, Emin Salimov, Robert Rieben, Nicolai Bovin

**Affiliations:** 1 Shemyakin-Ovchinnikov Institute of Bioorganic Chemistry Russian Academy of Science, 117991 Moscow, Russia; 2 National Medical Research Center for Obstetrics, Gynecology and Perinatology of the Ministry of Health of the Russian Federation, 117991 Moscow, Russia; 3 Zelinsky Institute of Organic Chemistry Russian Academy of Science, 119991 Moscow, Russia; 4 Branch of the Shemyakin-Ovchinnikov Institute of Bioorganic Chemistry of the Russian Academy of Sciences, 142290 Pushchino, Moscow Region, Russia; 5 School of Engineering, AUT University, Auckland 92006, New Zealand; 6 Federal Research Center for Original and Promising Biomedical and Pharmaceutical Technologies, 125315 Moscow, Russia; 7 Clinical Center of Sechenov First Moscow State Medical University of the Ministry of Health Care of the Russian Federation, 119435 Moscow, Russia; 8 Department for BioMedical Research, University of Bern, 3008 Bern, Switzerland

**Keywords:** printed glycan array, anti-glycan antibodies, complement, *O*-antigens, polysaccharides, kodecytes

## Abstract

**Background.** Naturally occurring human antibodies against glycans recognize and quickly eliminate infectious bacteria, viruses and aberrantly glycosylated neoplastic malignant cells, and they often initiate processes that involve the complement system. **Methods.** Using a printed glycan array (PGA) containing 605 glycoligands (oligo- and polysaccharides, glycopeptides), we examined which of the glycan-binding antibodies are able to activate the complement system. Using this PGA, the specificities of antibodies of the IgM and IgG classes were determined in the blood serum of healthy donors (suggested as mostly natural), and, then, using the same array, it was determined which types of the bound immunoglobulins were also showing C3 deposition. **Results.** It was found that about 30% of anti-glycan antibodies in human serum detected by the PGA did not activate the complement. They were mostly IgGs and directed to bacterial *O*-antigens; no apparent common structural motif within their target polysaccharides was found. Antibodies to blood group systems ABO and Forssman, xeno-antigens, a number of polysaccharides from various strains of *S. enterica*, *E. coli* and *P. alcalifaciens*, as well as small fragments of bacterial polysaccharides were recognized by complement-activating antibodies as expected. A complement-activating antibody was affinity-isolated on glycan-Sepharose from human serum, and, in the presence of the complement, it lysed red blood cells coated with the same glycan (kodecytes, where glycans expressed on biological membranes), while an isolated complement non-activating antibody did not, which confirms the validity of the solid-phase PGA results. **Conclusions.** Thus, ~30% of human anti-glycan antibodies lack the ability to activate the complement system. The function of the widely represented immunoglobulins that do not cause C3 deposition remains unclear.

## 1. Introduction

A common component involved in all three (classical, alternative and lectin) pathways of the complement system is the C3 protein, which is activated with the participation of the C3-convertase C4b2b in the classical and lectin pathways, or C3bBb in the alternative pathway [1,2]. As a result of proteolysis, C3 converts into C3a and C3b, the latter reacting with the Fab region of immunoglobulins but also binding to cell surfaces. C3b is then either split further (deactivated) by the contribution of factors H and I into the component C3d [2,3] or, if not inactivated, becomes part of the C5 convertase of the classical and lectin pathways C4b2b3b, or the alternative C5 convertase C3bBb3b, and complement activation can continue to the final stage of cell lysis via binding of C6–C9 and formation of the membrane attack complex C5b-9.

Antibodies are involved in all three pathways of complement activation: classical (by binding the C1q component with their Fc region), alternative (due to spontaneously formed C3b capable of attaching to immunoglobulin) [4] and, indirectly, lectin (due to interactions of mannan-binding lectin (MBL) with glycans present on IgG or IgA, but not IgM) [5,6,7,8]. C3b bound to immunoglobulins on the cell membrane interacts with the complement receptor on follicular dendritic cells and B cells (CR2 receptor), as well as macrophages (CR3 and CR4 receptors), activating them and facilitating the presentation of the recognized antigen [2,9,10].

Naturally occurring antibodies, i.e., immunoglobulins that appear in the organism without an obvious pathogen-specific stimulation of the immune system and make up the majority of antibodies in healthy human blood (which also includes the adaptive ones), are also actively involved in the complement cascade [11,12,13]. Antibodies to mono-, oligo- or polysaccharides, as well as to glycopeptides (anti-glycan antibodies, AGAs), a representative part of the total pool of natural antibodies, are no exception [12,13]. For example, it is well known that complement-mediated hemolysis can occur in the case of ABO-mismatched blood transfusion [14]. Upon xenotransplantation, antibodies to the so-called αGal antigen and Hanganutziu-Deicher, HD (Neu5Gcα2-3Galβ1-4Glc) antigen, trigger the complement cascade, causing acute tissue/organ rejection [15,16,17,18]. Anti-bacterial immunoglobulins directed towards rhamno-lipopolysaccharides and capable of activating the complement have been well studied [19]. It should be noted that very high titers of antibodies to the Rha monosaccharide are found in human blood [20,21]. To date, several hundred different specificities of anti-glycan antibodies are known [20,21,22]; however, whether all of them are capable of activating the complement system is unknown. In this work, using a printed glycan array containing 605 glycoligands (oligo- and polysaccharides, glycopeptides), we analyzed the ability of human blood AGAs (IgG and IgM) to initiate the deposition of C3 and investigated whether some of these antibodies can also lyse red blood cells (RBCs) in a complement-dependent manner (thus showing whether the solid-phase PGA results have some equivalence with what would happen on a biological membrane).

## 2. Materials and Methods

### 2.1. Serum Samples

Sera and red blood cells from healthy adult blood donors were obtained from the Sechenov First Moscow State Medical University Blood Center according to standard clinical and laboratory rules of the Russian Federation. All subjects gave written informed consent form approved by the local ethics committee of I.M. Sechenov First Moscow State Medical University (№ 07-17, 13 September 2017, Moscow, Russia). The characteristics of the donors are presented in Table 1.

### 2.2. PGA Assay

Printed glycan arrays (PGAs) in the form of a microchip were from GlycoNZ, Auckland, New Zealand. All inorganic salts were from (Sigma-Aldrich, USA). Chips were printed by robotic non-contact printer sciFLEXARRAYER S5 (Scienion, Berlin, Germany) using a drop volume of ~0.9 nL. 378 amino-spacered glycans and 227 bacterial *O*-polysaccharides in the print buffer (a 300 mM sodium phosphate, pH 8.5, containing 0.005% Tween-20 (Fluka, Buchs, Switzerland)) were deposited as six replicates onto *N*-hydroxysuccinimide-activated glass slides H (Schott Nexterion, Jena, Germany) at 20 µM and 2 µg/mL, respectively. A complete list of the printed glycans can be found in the Supplementary Material (Supplementary Material File S1). The purity of the glycans used was 95–97% according to NMR and HPLC. The NMR data of polysaccharides and related references are available in the polysaccharide database [23].

For the profiling of AGAs, the serum was diluted 1:15 in phosphate-buffered saline (PBS, pH 7.4) containing 1% Tween-20 and 1% BSA (Sigma-Aldrich, USA) (PBS-1% T20/1% BSA) and spun at 8000× *g* for 5 min. The supernatant obtained after this centrifugation was applied to the chip (pretreated with phosphate-buffered saline containing 0.1% Tween-20 (PBS-0.1% T20) for 15 min) and incubated in a humidified chamber at 37 °C for one hour with mild agitation (30 rpm). The PGA was then washed with PBS containing 0.05% Tween-20 (PBS-0.05% T20) and incubated with a mixture of Alexa555-labeled goat anti-human IgG and Alexa647-labeled goat anti-human IgM (cat. 21433 and 21249, respectively, Invitrogen, Carlsbad, USA) diluted 1:250 in PBS-1% T20 at 37 °C for one hour with mild agitation. The chip was washed with PBS-0.05% T20 followed by deionized water.

For C3 binding, the serum to be profiled was diluted 1:15 in PBS-1% T20/1%BSA, containing 0.5 mM of MgCl_2_ and 0.15 mM of CaCl_2_ (Fluka, Switzerland) or 5 mM of EDTA (Serva, Heidelberg, Germany), pre-heated at 37 °C for 15 min and spun at 8000× *g* for 5 min. The supernatant obtained after centrifugation was applied to the chip in a humidified chamber at 37 °C for one hour with mild agitation. The chip was washed with PBS-0.05%, and anti-C3 (a mixture of murine monoclonal anti-C3b and anti-C3d antibodies, Gamma-clone, Immucor, Norcross, GA, USA) was added and incubated at 37 °C for one hour with mild agitation. After washing with PBS-0.05% T20, biotinylated goat anti-mouse IgG + IgM(H + L) (cat. 31782, Thermo Fisher Scientific, Rockford, IL, USA) diluted 1:100 in PBS-1% T20/1% BSA was added, and the chip was incubated at 37 °C for one hour with mild agitation followed by a washing step with PBS-0.05% T20. Streptavidin–Alexa555 (Invitrogen, USA) diluted 1:1000 with PBS-0.1% T20) was applied to the chip, and, after incubation at 20 °C for one hour, the chip was washed with PBS-0.05% followed by deionized water.

Fluorescence signal intensities corresponding to the antibodies bound to glycoligands on the PGA were measured with a ScanArray Gx plus fluorescence scanner (PerkinElmer, Shelton, CT, USA) at a resolution of 10 µm. The images were processed using ScanArray Express 4.0 (fixed circle method) and then Microsoft Excel 2013 software. Signals were measured as the median relative fluorescence units (RFUs) within replicates and then normalized by the maximum signal value on each particular chip (normRFU). A fluorescence signal intensity, which exceeded the 25th percentile, was considered as significant. If the positive signal was detected for antibodies (IgG and/or IgM) and/or C3 deposition for a single donor, the glycoligand was designated as positive, while it was considered frequently encountered if reactive with samples of at least three of the five donors. Figures were plotted using Microsoft Excel software. The Spearman correlation coefficient was calculated by SPSS Statistics software, version 21.0 (IBM, Armonk, NY, USA). Comparisons between multiple groups were performed using a one-way ANOVA test (GraphPad Prism 8, GraphPad Software, Boston, MA, USA).

### 2.3. Affinity-Purified Antibodies

The affinity purification of two specific antibodies was performed using a BioLogic LP system with a flow spectrophotometric cell (BioRad, Hercules, CA, USA). A solution of 100 mL of pooled serum (consisting of sera from 156 healthy donors) was heat-inactivated for 30 min at 56 °C and centrifuged (7000× *g*, 20 min). A solution of 99 mL of supernatant was diluted 2.5 times with PBS (pH 7.4) containing 0.02% NaN_3_ and passed through a 2 mL column containing the adsorbent Galβ1→3GlcNAcβ−spacer−PAA−Sepharose 6FF (Le^C^-epitope) or Galβ1→4Glcβ←Gly←Leu-PAA−Sepharose 6FF (Lac-epitope) (GlycoNZ, New Zealand) at a flow rate of 24 mL/h. Before applying the pooled serum, the column was washed and equilibrated with PBS, containing 0.1% BSA (PBS/0.1% BSA). The ratio of total immunoglobulin per adsorbent volume was 0.75 g/mL. After perfusion with serum, the adsorbent was washed with PBS-0.1% T20 (20 mL) and then PBS (100 mL) until reaching zero absorbance of the effluent at 280 nm by the flow UV detector. Bound antibodies were eluted with 0.2 M of Tris-OH, pH 10.2, at a flow rate of 0.2 mL/min controlled by the flow spectrophotometric cell at 280 nm. Fractions containing antibodies were immediately neutralized to a final pH of 7.3–7.5 with 2 M of glycine (Sigma-Aldrich, USA) adjusted to pH 2.5 with HCl. After elution, the resulting volume was passed through a PAA−Sepharose 6FF column (GlycoNZ, New Zealand) to remove matrix-reactive immunoglobulins. This column was pre-washed and equilibrated with a Tris-glycine buffer (0.2 M of Tris-OH neutralized by adding 2 M of glycine−HCl to the final pH of 7.3–7.5). Purification was controlled using spectrophotometry at 280 nm until zero absorbance of the effluent. The recovered antibodies (affinity purified anti-Le^C^ and anti-Lac) were concentrated by centrifugation using Amicon Ultra Centrifugal Filter Unit 15 kDa cutoff (Merck, Darmstadt, Germany) at 4 °C, 5500× *g*. The final concentration of antibodies was determined by the Ultrospec 3100 pro spectrophotometer (Amersham, Little Chalfont, UK). All the buffers used contained 0.02% NaN_3_ (Sigma-Aldrich, St. Louis, MO, USA).

### 2.4. Preparation of Kodecytes

The terminology and methodology for describing functional-spacer lipid (FSL) constructs and the resultant kodecytes (cells labeled with FSL constructs) are described in detail elsewhere [24]. In brief, the protocol for kodecyte preparation involves mixing one volume of a solution of FSL constructs where F is Galβ1→3GlcNAcβ←CH_2_CH_2_NH_2_ (Le^C^-epitope) or Galβ1→4Glcβ←Gly←Leu (Lac-epitope) (GlycoNZ, New Zealand) in PBS at a 10 µM concentration with an equivalent volume of washed human RBCs (6% suspension in PBS) and incubating for one hour at 37 °C followed by washing thrice in PBS.

### 2.5. Flow Cytometry of Kodecytes

100 µL 3% suspensions of Le^C^-kodecytes and Lac-kodecytes and unmodified blood group A RBCs (as a negative control) in PBS were mixed with the same volume of the corresponding affinity purified anti-Le^C^ or anti-Lac solutions (5 or 10 µg/mL in PBS) and then stirred and incubated for one hour at 37 °C. After that, the samples were washed three times with PBS and subsequently incubated with a solution of goat anti-human IgM antibodies labeled with Alexa647 (cat. 21249, Invitrogen, USA) diluted 1:250 in PBS or goat anti-human IgG antibodies labeled with Alexa647 (cat. 109-606-170, JacksonImmunoResearch, West Grove, PA, USA) diluted 1:250 in PBS. Finally, samples were washed with PBS and resuspended in 1 mL of PBS. The stained cells were analyzed, and the fluorescence was measured using a Cytomics FC 500 flow cytometer (Beckman Coulter, Inc., Brea, CA, USA).

### 2.6. Hemolysis of Kodecytes

A 3% suspension of kodecytes (or unmodified RBC) was incubated with affinity-purified antibody samples (anti-Le^C^ at 5 μg/mL, or anti-Lac at 5 μg/mL or 10 μg/mL) at 37 °C for one hour. After washing 3 times with 1 mL of PBS, the cells and autologous (from the same donor as used for the red cells) human serum, as a source of complement, were incubated at 37 °C for one hour. The same serum containing 5 mM of EDTA (as a chelator of Ca^2+^/Mg^2+^ ions, required for complement activation) was used as a negative control. To define the contribution (if any) of potential natural anti-Le^C^ and anti-Lac present in the serum used as a source of complement, the hemolysis reaction with the same serum but without the addition of the affinity-purified antibodies was tested against the kodecytes. No hemolysis was observed, thus indicating that any natural anti-Le^C^ and anti-Lac that may have been present in the serum used as the source of complement did not cause the observed hemolysis. The incubation of native red cells with the anti-Le^C^ antibodies was also used to confirm the absence of their binding with RBCs. Hemolysis levels after incubation with serum were determined by spectrophotometry (absorbance at 405 nm).

## 3. Results

Using PGAs containing 378 glycans and 227 bacterial *O*-specific polysaccharides (*O*-antigens), the individual blood sera of five apparently healthy donors were analyzed.

Three sets of data were obtained for each donor: (1) the glycan-binding profile of IgG, as determined with fluorescently labeled anti-IgG; (2) the same for IgM, but with the anti-IgM secondary antibody; and (3) the deposition of C3 as determined using anti-C3.

In the presence of EDTA, almost all signals from C3 disappeared (see the “Control” in Supplementary Material File S1), which indicates the reliable detection of C3 deposition on the PGA with anti-C3 antibodies. The anti-C3 did not bind to the PGA glycans. If the donor had a signal above the cut-off level (25th percentile based on the exponential mode of the data distribution) in the IgG and/or IgM and/or C3 profile, the glycan was considered positive. If a particular AGA was found in three or more out of five donors, such a glycan was considered to be frequently encountered.

### 3.1. Characterization of Anti-Glycan Antibody and C3 Deposition Profiles

**Figure 1:**
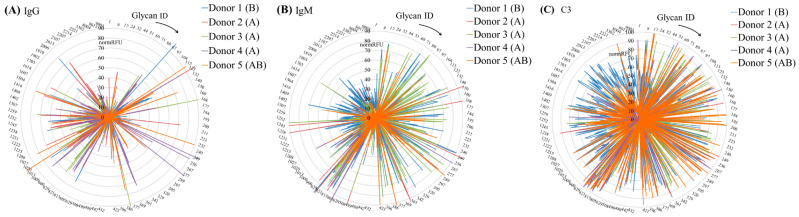
Comparison of anti-glycan IgG (**A**) and IgM (**B**) as well as C3 deposition profiles (**C**) for donors 1–5 (donor number and line color is indicated in the top right for each image, blood group is given in brackets). Data are presented as radar charts where the array data are sorted by the glycan ID (ID—identification number, see Supplementary Material File S1 for the full data; ligands with an ID that is more than 1000 are polysaccharides (the left part of the chart), less than 1000—mono and oligosaccharides (the right part of the chart)). In order to compare data, RFU values were normalized as % of the maximum signal on the chip (normRFU).

Comparing the profiles of AGAs, IgM antibodies were more diverse and were not always concordant with the IgG profile, which is consistent with the literature data [25]. The observed spectrum of specificities is close to those of previous studies of AGAs in human serum (Figure 1, full information is given in Supplementary Material File S1), [21,22,26,27].

**Figure 2:**
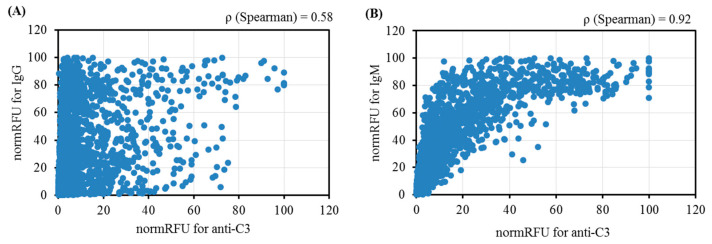
The relationship between anti-C3 and AGA binding activity of five donors (total data): (**A**) IgG antibodies, and (**B**) IgM antibodies.

The high signal for IgM antibodies in most cases also corresponded to a high signal for C3 deposition, whereas this was not the case for IgG (Figure 2). Spearman’s IgM:C3 deposition correlation coefficient for the total data from the five donors was 0.92, whereas it was 0.58 for IgG:C3 deposition.

Matching the profiles of AGAs and their ability to bind C3 on the PGA identified six major groupings (Table 2). Tri- and tetrasaccharide A and B glycans were excluded because there were insufficient samples for the data to be sorted with respect to ABO reactivity. It should be noted that a glycan was counted as an observation in a group (Table 2) if at least one of the five samples tested was positive.

**Figure 3:**
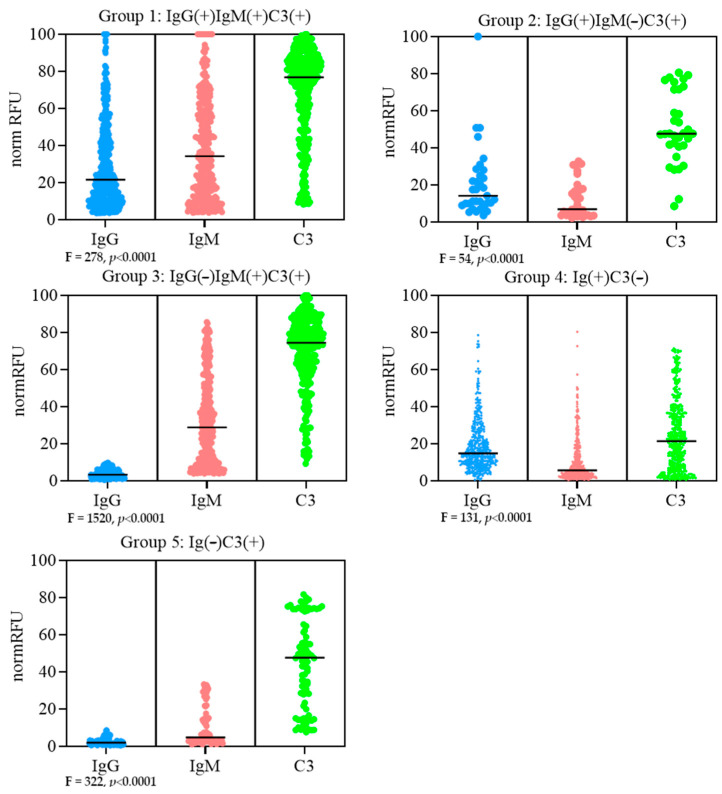
Comparison of signal intensity distributions of IgG and IgM-binding as well as C3 deposition for profile Groups 1–5. All bee-swarm plots are provided with the median. Data are shown for all glycoligands that meet the reactivity criteria of positivity for a minimum of one donor. *p* < 0.0001 for all groups. The ANOVA analysis revealed a substantial disparity between IgG/IgM/C3 signal intensities within all tested groups (*p* < 0.0001 in all cases).

An in-depth analysis of profiles of AGAs and their ability to deposit C3 on the PGA is as follows:

Group 1: IgG(+)IgM(+)C3(+): this group contains glycoligands interacting with both IgM and IgG antibodies and the deposition of C3 (Supplementary Material File S1). This group included 142 glycoligands (which meet the criteria of positivity for a minimum of one donor), of which 47 were positive with three or more of the five donors (Figure 3). The positive reactions were predominantly against bacterial *O*-polysaccharides from several strains of *S. enterica*, *E. coli*, *P. alcalifaciens* and *S. boydii*, among which are the PS carrying *L*-Rhaα or GlcNAcα residues as side substituents (note *L*-Rhaα and GlcNAcα as monosaccharides are also included in this group); other bacterial polysaccharide fragments such as glucosaminylmuramyl peptide (GMDP-Lys), the trisaccharide KDOα, the disaccharide fragment Galα1-4GlcNAcβ of *Bacillus anthracis* glycoprotein, and the following oligosaccharides: Forssman pentasaccharide and its fragments, melibiose.

Group 2: IgG(+)IgM(−)C3(+): this group of glycoligands show significant interaction with IgG (but not IgM) antibodies and the deposition of C3 (Supplementary Material File S1). This group includes 29 glycans (Figure 3), and the frequency of reactivity was mostly low (one or two positives out of five donors). The only exception was pentasaccharide Galα1-3Galβ1-4GlcNAcβ1-3Galβ1-4Glcβ (αGal antigen), for which significant signals for both IgG and C3 were detected in at least three of the five donors.

Group 3: IgG(−)IgM(+)C3(+): this group of glycoligands shows a significant interaction with IgM (but not IgG) antibodies and with C3 deposition (Supplementary Material File S1). This group includes 146 glycans (Figure 3), of which 45 react with at least three out of five donors. This group included the sulfated disaccharides 4′-*O*-Su- and 4′,6′-*O*-Su_2_-Galβ1-4GlcNAcβ, 3′-*O*-Su- and 3′,6′-*O*-Su_2_-GalNAcβ1-4GlcNAcβ; neutral disaccharide Galβ1-3GlcNAcβ (Le^C^) and structurally related glycans (according to [28]); core fragments of *O*-chains (core 3, core 5 and other glycans with GalNAcα at the reducing end), fragments of glycolipids (P1, Gb4) and unnatural Neu5Acβ-containing saccharides. *O*-PS in the profiles of this group are practically absent.

Group 4: Ig(+)C3(−): this group in which IgM and/or IgG are detected but with no C3 deposition contained 203 glycoligands—about 30% were detected by the PGA (Supplementary Material File S1, Figure 3), 66 of which bound immunoglobulins in at least three out of the five donors, and these were usually IgG(+). The antibody reactive glycans in this group are *O*-PS from *E. coli*, *S. enterica*, *P. aeruginosa* and *S. flexneri*, as well as the monosaccharide Galβ and amino acid derivatives of melibiose, lactose and oligosaccharides X→Galβ1→4GlcβNH←peptide (where X is H, Neu5Acα2-3 or Galβ1-4GlcNAcβ1-3). Ten *O*-polysaccharides from *E. coli*, *S. enterica*, *P. aeruginosa*, and *S. flexneri* (in addition to those mentioned above) interacted with both IgM and IgG. Against *E. coli* O148 *O*-PS, only IgM reactions were detected. No apparent common structural motif was noted for this group of glycoligands.

Group 5. Ig(−)C3(+): this group contained glycoligands, which, despite the absence of detectable antibody interactions, C3 deposition was observed (Supplementary Material File S1). This group consisted of 70 glycoligands (Figure 3), against which only occasional antibodies were seen in one or two donors. The significant anti-C3 signals were observed for *O*-polysaccharide from *E. coli* O52 (four individuals) and hyaluronic acid (three individuals), respectively. The contribution of these positive C3 results (if any) to the result in Groups 1, 2 and 3 is unknown.

Group 6: Ig(−)C3(−): this group contained 474 unreactive glycoligands (Supplementary Material File S1); 220 were unreactive with all five donors and 370 were unreactive with at least three of five donors. As expected, many of the unreactive glycans were those that are components of mammalian cells, against which antibodies are not expected in healthy donors [22,27]; this group included the Neu5Acα-terminated glycans and oligosialic acid, sialylated complex type bi-antennary N-chain and its desialylated and degalactosylated variants. Other glycans belonging to this group are some bacterial *O*-PS and plant PS (laminarin, glucans and galactans).

### 3.2. Complement-Mediated Cell Lysis Test Confirms Functionality of Complement-Activating AGA

Group 4: (Ig(+)C3(−) (Table 2) consists of glycans to which antibodies react yet do not cause complement deposition, which suggests that some may lack the ability to activate the complement. In order to test the observed effect under conditions closer to the natural situation, the possibility of the complement-mediated hemolysis of red cells whose surface was modified with the corresponding glycans (in the form of synthetic glycolipids, creating so called kodecytes [24]) were studied. Affinity-purified antibodies were isolated from the pooled serum using hapten-specific affinity chromatography [29,30]. The first sample of antibodies, anti-Lac from Group 4 (where the binding of C3 was undetected), was isolated using an adsorbent with Galβ1→4Glcβ←Gly←Leu glycan. The second sample, anti-Le^C^ from Group 3 (IgG(−)IgM(+)C3(+)), was isolated on an adsorbent with Le^C^ disaccharide. Antibodies to Le^C^ circulate in the blood of most donors [21] and were chosen as a positive control because of their ability to bind the complement according to the results of the PGA.

**Figure 4:**
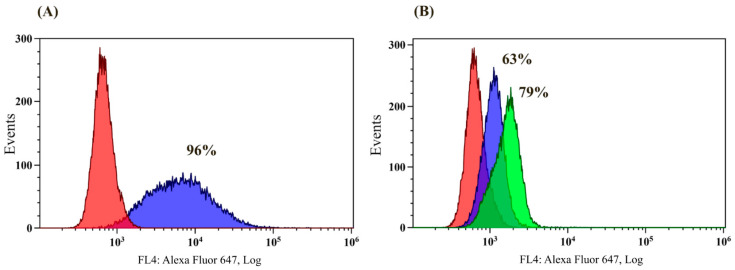
The interaction of affinity-purified anti-Le^C^ and anti-Lac with Le^C^- and Lac-kodecytes. Flow cytometry data (Cytomics FC500 Beckman Coulter). (**A**) Le^C^-kodecytes with anti-Le^C^. Red zone, negative control (Le^C^-kodecytes, no anti-Le^C^). Blue zone, Le^C^-kodecytes with anti-LeC (5 µg/mL), 96% positive cells. (**B**) Lac-kodecytes with anti-Lac. Red zone, negative control (Lac-kodecytes, no anti-Lac). Blue zone, Lac-kodecytes with 5 µg/mL of anti-Lac, 63% positive cells. Green zone, Lac-kodecytes with 10 µg/mL of anti-Lac, 79% positive cells. The percentage of positive cells was obtained by the method of channel-by-channel subtraction.

To confirm the interaction of anti-Lac and anti-Le^C^ with corresponding kodecytes, a cytofluorimetric analysis was performed. Detection of the antibodies bound to the kodecytes was performed using secondary immunoglobulins specific for human IgM in case of anti-Le^C^ (because it was shown that these antibodies belong mainly to the IgM class [31]) and specific to IgG in case of anti-Lac since these classes of immunoglobulins were those detected for this glycan in the PGA. No binding of isolated antibodies or secondary antibodies to the native RBCs was observed. The binding of anti-Le^C^ (5 µg/mL) to Le^C^-kodecytes was 96%, while 5 µg/mL of anti-Lac binding to Lac-kodecytes was 63%, which increased to 79% for 10 µg/mL of anti-Lac (Figure 4).

**Figure 5:**
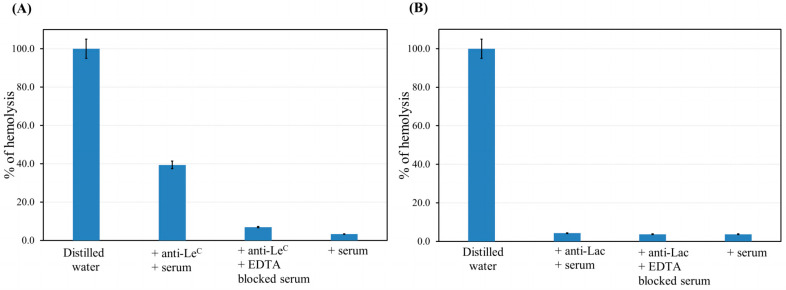
The ability of anti-Le^C^ (5 µg/mL) and anti-Lac (10 µg/mL) to activate the complement in the hemolytic test with kodecytes bearing Le^C^ (**A**) or Lac (**B**) antigens, respectively. Averaged data with standard deviations from two experiments are given.

After proving the Le^C^- and Lac-kodecytes were appropriately antigen–antibody reactive, they were incubated with an autologous serum (i.e., the serum of the same donor whose red cells were used) as a source of complement, and the degree of hemolysis was detected by measuring released hemoglobin. The same serum, in which the complement activity was blocked by EDTA, was used as a control. A non-complementary pair of kodecyte–antibody (Lac-kodecytes plus anti-Le^C^ antibodies) was also taken to ensure that the reaction (in this case, no reaction) was due to a specific antigen–antibody pair. Additionally, the serum used as the complement source was tested to confirm that it did not cause significant kodecyte hemolysis in the absence of anti-Le^C^ or anti-Lac. Affinity-purified anti-Le^C^ at a concentration of 5 μg/mL was able to induce the hemolysis of the corresponding Le^C^—kodecytes at a significant level (Figure 5A). At the same time, the intensity of the cell hemolysis of native (no inserted glycan) red cells, either in the presence of EDTA or in a mismatched kodecyte–antibody pair, was negligible, indicating that the hemolysis observed is primarily caused by the cognate antibodies (rather than eryptosis). In contrast, anti-Lac, even at a concentration of 10 µg/mL, did not activate the complement system and cause hemolysis (Figure 5B).

Thus, anti-Le^C^, which was defined as a complement-activating antibody (Group 3 IgG(−)IgM(+)C3(+)) in the solid-phase PGA, was confirmed to have this characteristic when tested against cells, in terms of inducing hemolysis. Similarly, anti-Lac, which was defined by the PGA as not complement activating (Group 4 Ig(+)C3(−)), did not lyse red cells. Although only two representative antibodies were tested, these results support the concept that PGA results should have some correlation with expected reactivity at cell membranes.

## 4. Discussion

Naturally occurring anti-glycan antibodies are widely present in human serum, which is evident from several studies using the printed glycan array (PGA) [20,21,26,32]. To date, the number of AGAs is over several hundred and continues to grow, as does the number of glycoligand probes in new versions of PGAs. However, only a few AGAs are well characterized, and, for the majority, there is at best only a statement of their ability to bind a corresponding glycan [14,16,17,18,29,30,31,33,34]. The supposed main function of AGAs is the recognition and removal of “foreign” or “altered” cells or particles as part of the innate immune system [10,22,35]. IgM is the primary molecule involved in the response (through the complement activation), although IgG (with the exception of IgG4) may also participate [36]. Despite the evidence of antibody interactions, the question remains whether all AGAs are capable of activating the complement system. To evaluate this, we used PGAs to determine if the AGAs recognizing specific glycans are also able activate the complement, by detecting the deposition of C3 (C3b and/or C3d) onto the PGA.

### 4.1. Characterization of Complement-Binding IgG and IgM

Complement-binding AGAs of Groups 1–3 (Table 2) are mainly IgM, with a high correlation between antigenicity and complement activation, while, for IgG, there is no such dependence (Figure 2). Moreover, in Groups 2 and 3, the repertoire of IgG complement-activating glycans is notably different from those that are IgM complement-activating. However, a number of glycans (included in Groups 1–3) are found, to which antibodies are present in all examined donors (Table 3, Supplementary Material File S1).

As can be seen in Table 3, those glycans that show significant interactions with immunoglobulins and activate the complement are bacterial polysaccharides or oligosaccharides generally not found in mammals. If the criteria is relaxed from five to at least three donors being positive, then the list of glycoligands in this group will increase to more than 60 glycoligands and will include, in particular, *L*-Rhaα, GMDP-Lys, blood group B and Forssman antigens, and other oligosaccharides (corresponding antibodies are considered natural [20,21,22]), as well as *O*-polysaccharides from a number of strains of *E. coli* and *S. boydii* (whose corresponding antibodies are most likely adaptive in origin).

It should be noted, that, although complement deposition does not affect the recognition of immunoglobulins by secondary antibodies (Supplementary Material File S2), we cannot exclude the possibility of the competitive/additive binding of immunoglobulins and serum lectins (including via an alternative activation pathway, see below) with glycoligands. The individual contribution of each of the components listed above cannot be determined with the experimental design used.

### 4.2. Complement Fixation in the Absence of Antibody Binding

**Figure 6:**
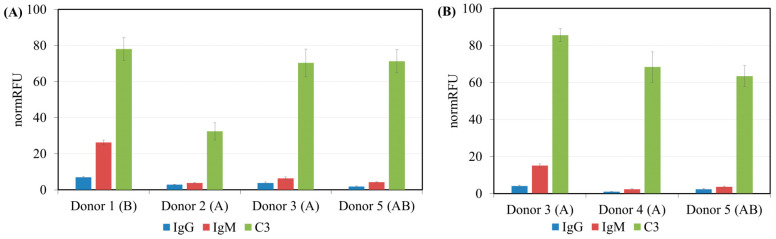
Distribution of binding of IgG, IgM and anti-C3 antibodies to *E. coli* O52 *O*-polysaccharide (**A**) and hyaluronic acid (**B**), belonging to group 5 Ig(−)C3(+), in donors meeting the criteria of this group. Donors’ blood groups are given in brackets.

Despite the fact that most PGA glycoligands bound both the AGA and complement, a small group (Group 5 Ig(−)C3(+)) was found in which immunoglobulins did not appear (or at least were not detected by the PGA) to activate the complement but still showed significant signals when detecting C3 deposition. Two examples of such glycans were the *O*-polysaccharide from *E. coli* O52 and hyaluronic acid (Figure 6). Thus, there are glycoligands that fix the complement independent of antibodies.

We consider the following possible reasons for the observed antibody-independent complement fixation:

- The presence of IgA antibodies in the blood serum (the used test system did not allow their detection). According to the literature, IgA itself does not activate the complement via the classical pathway, but it is able to interact with MBL, which, in turn, activates the lectin pathway of the complement system [7]. The preferred IgA targets are known to be polysaccharides [38,39], of which, in Group 5, there were up to a third of all glycoligands (see Supplementary Material File S1).

The direct interaction of MBL (as opposed to IgA-mediated) with PGA glycoligands is evidenced by the interaction of anti-C3 with the manno-oligosaccharide in the absence of interaction with IgG/M (Supplementary Material File S1). The MBL is known to be present in the blood of healthy donors at a level of several µg/mL [40,41] (i.e., at a level comparable to AGAs [22]) and is able to selectively recognize bacteria by activating the complement [42]. Moreover, with the help of PGAs, it was previously found that, in addition to manno-motifs, MBL is able to interact with GlcNAcβ-terminated, fucose-containing and some other glycans (http://functionalglycomics.org, study 1013 accessed on 14 June 2007), which is consistent with the repertoire of glycans included in Group 5 (Ig(−)C3(+)). The presence of MBL in the sera of the donors (except donor 2; see Supplementary Material File S2), as well as the interaction of anti-C3 with manno-oligosaccharide in the absence of interaction with IgG/IgM, is consistent with the proposed contribution of this MBL to the observed C3 deposition. Moreover, the complement can be activated by other lectins presented in the blood, such as ficolins, CL-K1, CL-L1 and CL-P1, and each is capable of recognizing bacterial polysaccharides [43,44].

### 4.3. Anti-Glycan Antibodies That Did Not Activate Complement

Some of the anti-glycan antibodies (Group 4 Ig(+)C3(−)) did not activate the complement when binding to glycoligands on the PGA. Because this is significantly different from in vivo conditions, we performed additional experiments using antibodies isolated by hapten-specific affinity chromatography from blood, namely, antibodies to the Galβ1-4GlcβNH←Gly←Leu glycopeptide (anti-Lac) and to the disaccharide Le^C^ (anti-Le^C^). The choice of these antibodies was due to their high occurrence in donors [21,22], as well as their classification into fundamentally different groups, namely, anti-Lac to Group 4 (found in three out of five donors studied), and anti-Le^C^ to Group 3 IgG(−)IgM(+)C3(+) (five out of five donors). The latter is a suitable positive control. The complement-mediated hemolysis of red blood cells modified with synthetic glycolipid (kodecytes, [24]) was carried out. As expected, the kodecytes were hemolyzed by affinity isolated anti-Le^C^ in the presence of serum as a source of complement, whereas no hemolysis was observed in the presence of anti-Lac (Figure 5). Thus, the hemolysis data confirmed the results of the PGA and provide grounds to consider other PGA results to be adequate.

Group 4 Ig(+)C3(−) antibodies belong predominantly to IgG, which, as it is known, attracts C1q only in the form of oligomeric immunoglobulin. The most likely reason for the observed abolition of the interaction is the suboptimal density/presentation of glyco-epitopes for complement binding, which nevertheless does not abolish antibody binding. Similarly, the interaction of class M immunoglobulins occurred only with some of *O*-polysaccharides; presumably, for a strong C1q interaction to occur, the IgM molecule adopts a “table” conformation [45]. There are other possible reasons why IgGs do not activate complement; below are three of those discussed in the literature. First: Lee et al. showed that it is possible to construct an IgG molecule capable of selectively interacting with C1q, but not with one of the FcγR, by just two amino acid substitutions (K320E in the Cγ2 domain and Q386R in Cγ3 of Fc-region) [46]; one can also expect the opposite situation, when the Fc fragment is organized to recruit an FcR only. Second: glycosylation, in particular, the fucosylation of IgG *N*-glycans, regulates the efficiency of interaction with the Fc receptor [47,48], without significantly affecting antigen-binding activity. Third: subtypes of IgG, namely, the presence of IgG4, which is not able to activate the classical pathway due to its structural features [49] but is able to interact with FcγR [50].

We see the paradox of the existence of Group 4 Ig(+)C3(−) antibodies in the fact that the absence of complement fixation is demonstrated for antibodies of only some specificities; that is, the effector properties are directly dictated by antigen specificity, which, according to generally accepted ideas, should not be the case. Although such phenomena have been observed—for example, when studying the effects of using the monoclonal antibodies recognizing overlapping epitopes of CD20 (rituximab, tositumomab and others), no explanation has yet been proposed for them [51]. The comparison of glycans, antibodies to which (Group 4) have such properties, does not reveal structural features common to all of them. Although a significant part (not all) of glycans are bacterial polysaccharides, belonging to them in itself does not provide the key to understanding, since antibodies to other polysaccharides do not exhibit such properties. The specific antigen presentation on the surface of the slide in the glycoarray is unlikely to be the reason, since, for one of antigens, Galβ1-4GlcβNH←Gly←Leu (see above), the same inability to fix the complement manifested itself when the corresponding lipophilic derivative was incorporated into the cell membrane. Thus, we do not yet have either an experimentally substantiated or speculative explanation for the observed phenomenon.

## 5. Conclusions

Among naturally occurring human antibodies against glycans (examined by a PGA, which has 605 ligands), approximately 30% were found to be glycan-binding but complement-inert, with a significant portion of them as top ligands in terms of IgG binding (normRFUs are up to 78, see Group 4 in Figure 3 and Supplementary Material File S1). Such a wide repertoire, as well as confirmation of the observed properties in an alternative (cellular) assay, suggests a general nature of the phenomenon. Therefore, it seems reasonable to assume that some of them perform specific functions, such as blocking (masking) antigens in allergy [52,53] or pregnancy [54], to abolish the effector function, thus preventing inflammatory reactions.

## Figures and Tables

**Table 1:** Characteristics of the donors.

Donor	Sex	Age	Blood Type
1	F	41	B
2	M	56	A
3	F	32	A
4	M	31	A
5	F	40	AB

**Table 2:** Profile groups of AGAs and complement deposition activity. All reactive glycoligands that met the criteria with at least one sample, as well as frequently encountered with at least three samples, are noted. A total of 605 glycoligands were printed onto the array.

	PGA Reactivity			Glycoligand Observations in ≥1 Sample (% From Total Glycoligands)	Glycoligand Observations in ≥3 Samples (% From Total Glycoligands)	Comments
Group	IgM	IgG	C3			
1	+	+	+	142 (23.5)	47 (7.8)	IgG(+)IgM(+)C3(+): Glycoligands that interact with both IgM and IgG antibodies and are complement-activating
2	−	+	+	29 (4.8)	1 (0.2)	IgG(+)IgM(−)C3(+): Glycoligands that interact with IgG (but not IgM) antibodies and are complement-activating
3	+	−	+	146 (24.1)	45 (7.4)	IgG(−)IgM(+)C3(+): Glycoligands that interact with IgM (but not IgG) antibodies and are complement-activating
4	**±**	**±**	−	203 (33.6)	66 (10.9)	Ig(+)C3(−): Glycoligands that interact with IgG and/or IgM antibodies but are not complement-activating
5	−	−	+	70 (11.6)	2 (0.3)	Ig(−)C3(+): Complement-activating glycoligands despite lack of detectable antibody binding
6	−	−	−	474 (78.3)	370 (61.1)	Ig(−)C3(−): Glycoligands that do not show the binding of IgG/IgM and are also complement-non-activating

**Table 3:** Glycans that show significant interactions with immunoglobulins and activate complement in all five donors.

Glycan ID	Structure	Class of Detected Immuno-Globulins
Mono- and oligosaccharides		
10	GlcNAcβ	IgM
85	Galβ1-3GlcNAcβ	IgM
154	3-*O*-Su-Galβ1-3GlcNAcβ	IgM
246	GlcNAcβ1-2Galβ1-3GalNAcα	IgM and IgG
820	GlcNAcβ1-4GalNAcα	IgM and IgG
378	Galβ1-3GlcNAcα1-3Galβ1-4GlcNAcβ	IgM and IgG
410	Galβ1-3GlcNAcα1-3Galβ1-3GlcNAcβ	IgM and IgG
813	Galβ1-3GalNGcα	IgM and IgG
825	Glcβ1-3GlcNAcβ	IgM and IgG
827	Glcβ1-3GalNAcβ	IgM and IgG
826	Glcβ1-3GalNAcα	IgM and IgG
815	Galα1-4GalNAcα	IgM and IgG
808	Galα1-6Glcβ	IgM and IgG
101	GalNAcα1-3GalNAcβ	IgM and IgG
818	GalNAcα1-4GalNAcα	IgM and IgG
817	GalNAcβ1-4GalNAcα	IgM and IgG
19	ManNAcβNH←Gly *	IgM and IgG
Repeating unit of *O*-antigen (strain)		
1008	-3Galf2(30%)Acβ1-3Galα1- (*S. enterica* O67)	IgM and IgG
1021	-3(GlcNAcβ1-2)Rhaα1-2Rhaα1-4Glcα1-3GalNAcβ1- (*S. enterica* O57 **)	IgM and IgG
1205	-3(GlcNAcβ1-2)Rhaα1-2Rhaα1-4Glcα1-3GalNAcβ1- (*E. coli* O51)	IgM and IgG
1308	-3(Dhpα2-4Manβ1-4)Galα1-4GalNAcβ1-3GalNAcβ1- (*P. alcalifaciens* O31)	IgM and IgG
1312	-4Glcβ1-3Galα1-4GalNAcβ1-4(L-Ser2-6)GlcAβ1-3GalNAcβ1- (*P. alcalifaciens* O60)	IgM and IgG

* Fucose and rhamnose are *L*-sugars by default, and all others are *D*-sugars. Structures of polysaccharides are shown in “glycan form” for convenience of comparison with oligosaccharides, meaning that designations of ring size (pyranose/furanose) and, in most cases, configuration (*D/L*) are omitted. Other abbreviations used are according to the polysaccharide database [23]. ** *S. enterica* O57 and *E. coli* O51 have the same repeated unit structure [37].

## Data Availability

The original data presented in this study are included in Supplementary Material. Further inquiries can be directed to the corresponding author.

## Supplementary Materials

The following supporting information can be downloaded at https://www.mdpi.com/article/10.3390/antib13040105/s1, Supplementary Material File S1; Supplementary Material File S2.
