# Supplementary material for: Some Human Anti-Glycan Antibodies Lack the Ability to Activate the Complement System
Source: Antibodies (Basel). 2024 Dec 23;13(4):105. doi: 10.3390/antib13040105 (PMC11672820; doi:10.3390/antib13040105)
Supplement: Supplementary file 1 [file antibodies-13-00105-s001.zip › Supplementary_Material File S2_f.pdf]

Supplementary Material 2

Some Human Anti-Glycan Antibodies Lack the Ability to Activate the Complement System

Nadezhda Shilova [1,2,*], Alexey Nokel [1,2], Alexander Lipatnikov [1], Nailya Khasbiullina [2], Yuri Knirel [3], Ludmila Baidakova [4], Alexander Tuzikov [1], Sergei Khaidukov [1,2], Polina Obukhova [1,2], Stephen Henry [5], Batozhab Shoibonov [6], Emin Salimov [7], Robert Rieben [8] and Nicolai Bovin [1]

[1] Shemyakin-Ovchinnikov Institute of Bioorganic Chemistry Russian Academy of Science, 117991 Moscow, Russia; anokel@internet.ru (A.N.); alex.9508@yandex.ru (A.L.); alextuzikov@yandex.ru (A.T.); khsergey54@mail.ru (S.K.); anruma@yandex.ru (P.O.); professorbovin@yandex.ru (N.B.)
[2] National Medical Research Center for Obstetrics, Gynecology and Perinatology of the Ministry of Health of the Russian Federation, 117991 Moscow, Russia; crosbreed@list.ru
[3] Zelinsky Institute of Organic Chemistry Russian Academy of Science, 119991 Moscow, Russia; knirel@gmail.com
[4] Branch of the Shemyakin-Ovchinnikov Institute of Bioorganic Chemistry of the Russian Academy of Sciences, 142290 Pushchino, Moscow Region, Russia; lionessa7591@gmail.com
[5] School of Engineering, AUT University, 92006 Auckland, New Zealand; stephen.henry@aut.ac.nz
[6] Federal Research Center for Original and Promising Biomedical and Pharmaceutical Technologies, 125315 Moscow, Russia; shoibonov@mail.ru
[7] Clinical Center of Sechenov First Moscow State Medical University of the Ministry of Health Care of the Russian Federation, 119435 Moscow, Russia; dc13@mail.ru
[8] Department for BioMedical Research, University of Bern, 3008 Bern, Switzerland; robert.rieben@unibe.ch
* Correspondence: pumatnv@gmail.com

1 The effect of complement on the interaction of blood immunoglobulins with secondary antibodies Introduction

1.1 Method

All inorganic salts were from (Sigma-Aldrich, USA).

MaxiSorp microtiter plates (NUNC, Denmark) were coated with glycan–PAA conjugates (GlycoNZ, New Zealand) or glycan-free PAA (as a background control), 10 µg/ml (100 µl/well), in the 50 mM sodium carbonate buffer (pH 9.6) for one hour at 37°C. The plates were blocked with PBS/1% BSA, 150 µl/well, for one hour at 37°C. 200 µl of a serum with and without 5 mM EDTA were added in the wells of the first line of the plate, all other wells were filled with 100 µl PBS/0.3% BSA. The samples were two-fold diluted and incubated for one hour at 37°C. The plates were washed with PBS-0.1% Tween-20 four times. HRP-labeled anti-human (IgM+IgG+IgA) secondary antibodies (Southern Biotech, USA), diluted 1:4000 with PBS/0.3% BSA, was added to the plates, 100 µl per well, and the plates were incubated for one hour at 37°C. The plates were washed with PBS-0.1% Tween-20 four times. Color was developed by a 15 min incubation at room temperature with 0.1 M sodium phosphate / 0.1 M citrate buffer containing 0.04% of o-phenylene diamine (Sigma-Aldrich, USA) and 0.03% of H_2O_2, 100 µl per well. The color reaction was stopped by the addition of 30 µl 1 M H_2SO_4. The absorbance was read at 490 nm (0.1 s) with a plate reader

Wallac1420 Multilabel Counter, Victor2 (Perkin Elmer Life Sciences, Finland). All the tests were performed in duplicate; the differences between readings (intra-assay) did not exceed 5%.

1.2 Result

AGA profiling is performed on blood serum samples that contain complement proteins, which can directly interact with the array's glycoligands, or bind to AGA in the process of recognition of glycans (what we really observe, see the results above and below). In the last case, complement bound to the AGA distorts the pattern of standard AGA profiling via influence on interaction of the secondary (labelled) antibodies. To find out whether such effects take place, we investigated the interaction of blood serum in the presence and absence of EDTA, the reagent abolishing the binding of complement proteins, using the example of the disaccharide Le^C (included in group №3) in the ELISA format. It turned out that EDTA does not affect the binding of secondary antibodies to either IgG or IgM of anti-Le^C specificity (Supplementary Figure 1).

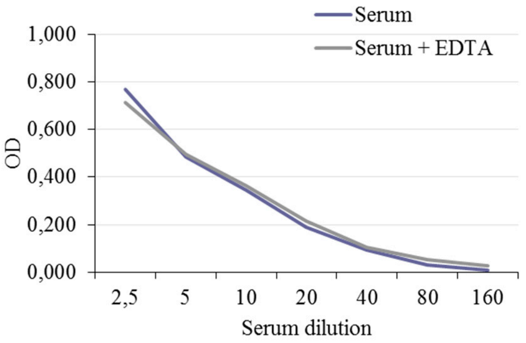

(A)

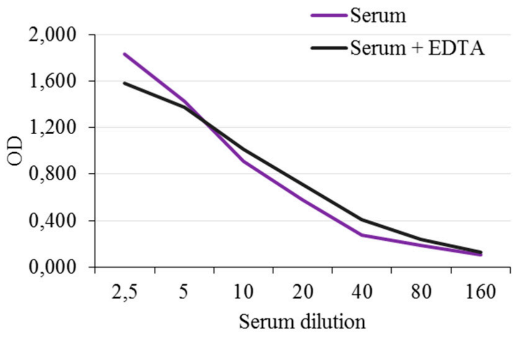

(B)

Supplementary Figure 1. Interaction of IgG (A) and IgM (B) of the donor's serum and blood plasma with LeC (as a polyacrylamide conjugate, PAA) using secondary antibodies. Data are provided minus background (signal from wells with glycan-free PAA).

2 Activity of the lectin pathway of complement.

2.1 Method

Activation of the lectin pathway of complement was performed by using the commercial kit Complement System MBL (Euro Diagnostica, Sweden) according to the manufacturer's instructions. This assay based on measuring of activity of monoclonal antibodies specific for a neoepitope of the terminal complement complex, C5b-9, produced as a result of MBL complement activation. Serum with known MBL-activity was use as a standard (included to the kit).

2.2 Result

It is necessary to explain the presence of glycoligands, for which an interaction with the C3b/C3d complement system proteins was observed, despite the absence of any AGA binding to the array. A possible reason is the activation of the lectin pathway, primarily through the mannose-binding lectin (MBL), which is able to independently interact with both array glycans and oligosaccharide residues of bound anti-glycan IgA (see https://doi.org/10.4049/jimmunol.167.5.2861), which we did not detect in these experiments.

To determine the possible involvement of MBL, an enzyme immunoassay was performed to estimate the presence of this lectin in the donor’s sera. It was found that only one donor had no MBL (Supplementary Figure 2), while its content in serum of the rest donors was comparable to that in standard serum, which is consistent with the assumption about the contribution of lectin pathway to the observed results.

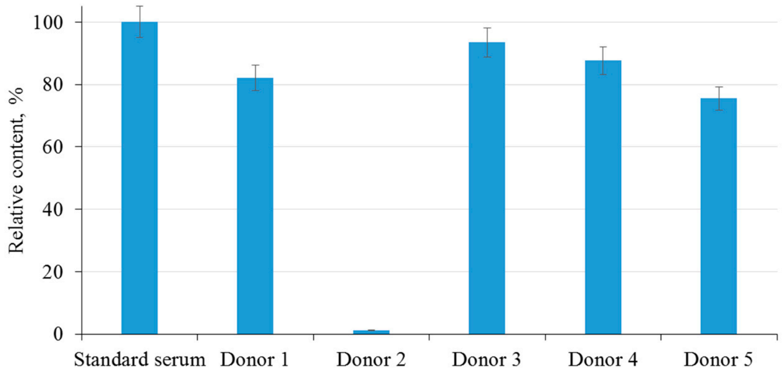

Supplementary Figure 2. The content of mannose-binding lectin (relative to standard serum with known lectin pathway activity, %) in the studied donors (see the Table 1 in the main text for the description). Averaged data for three experiments are given with standard deviations.
